# Sustained intracellular calcium rise mediates neuronal mitophagy in models of autosomal dominant optic atrophy

**DOI:** 10.1038/s41418-021-00847-3

**Published:** 2021-08-13

**Authors:** Marta Zaninello, Konstantinos Palikaras, Aggeliki Sotiriou, Nektarios Tavernarakis, Luca Scorrano

**Affiliations:** 1 Veneto Institute of Molecular Medicine, Padova, Italy; 2 Department of Biology, University of Padova, Padova, Italy; 3 Department of Physiology, School of Medicine, National and Kapodistrian University of Athens, Athens, Greece; 4 Institute of Molecular Biology and Biotechnology, Foundation for Research and Technology-Hellas, Heraklion, Crete Greece; 5 Department of Basic Sciences, School of Medicine, University of Crete, Heraklion, Crete Greece; 6 Present Address: Cologne Excellence Cluster on Cellular Stress Responses in Aging‐Associated Diseases (CECAD), University of Cologne, Cologne, Germany

**Keywords:** Cell biology, Neurological disorders

## Abstract

Mitochondrial dysfunction and mitophagy are often hallmarks of neurodegenerative diseases such as autosomal dominant optic atrophy (ADOA) caused by mutations in the key mitochondrial dynamics protein optic atrophy 1 (Opa1). However, the second messengers linking mitochondrial dysfunction to initiation of mitophagy remain poorly characterized. Here, we show in mammalian and nematode neurons that Opa1 mutations trigger Ca^2+^-dependent mitophagy. Deletion or expression of mutated Opa1 in mouse retinal ganglion cells and *Caenorhabditis elegans* motor neurons lead to mitochondrial dysfunction, increased cytosolic Ca^2+^ levels, and decreased axonal mitochondrial density. Chelation of Ca^2+^ restores mitochondrial density in neuronal processes, neuronal function, and viability. Mechanistically, sustained Ca^2+^ levels activate calcineurin and AMPK, placed in the same genetic pathway regulating axonal mitochondrial density. Our data reveal that mitophagy in ADOA depends on Ca^2+^-calcineurin-AMPK signaling cascade.

## Introduction

Neuronal health crucially depends on mitochondria, which supply most of the ATP required for their function and growth. Not surprisingly, neurodegeneration is often associated with mitochondrial dysfunction [1–4]. In the last decade, the basic tenets of mitophagy, the removal of mitochondria by autophagy, have been elucidated. The core components of the mitophagy machinery are linked to genetic forms of neurodegeneration: the Parkinson’s disease genes PTEN-induced kinase 1 (PINK1; PARK6) and the cytosolic E3 ubiquitin ligase Parkin (PARK2) are centrally placed in the mitophagy pathway [5–7]. In the most parsimonious model, mitochondrial dysfunction leads to membrane depolarization, impaired import of PINK1 that once imported in healthy cells is constitutively degraded by the inner mitochondrial membrane presenilin-associated rhomboid such as (PARL) protease, PINK1 accumulation on the outer mitochondrial membrane, and recruitment and stabilization of Parkin that ubiquitinates promiscuous substrates on the OMM to flag these damaged organelles for engulfment by the nascent autophagosomes [5–7].

While the PINK1–Parkin pathway efficiently eliminates dysfunctional mitochondria in several cell types, neurons often show delayed Parkin-mediated mitophagy in response to mitochondrial depolarization [8–10]. Indeed, at a major difference from flies, in Parkin or PINK1 deficient mice mitochondrial morphology and neuronal health are largely unaffected [11]. These findings suggest that other mechanisms than the finely regulated Parkin-mediated mitophagy operate to maintain mitochondrial integrity in neurons and neuronal health upon mitochondrial dysfunction. Indeed, inhibition of autophagy paradoxically protects retinal ganglion cells (RGCs) from the consequences caused by expression of mutants of the key mitochondrial dynamics gene optic atrophy 1 (Opa1), mutated in the neurodegenerative disease autosomal dominant optic atrophy (ADOA), and corrects the visual defect caused by conditional RGC Opa1 ablation. Similarly, autophagy inhibition protects *Caenorhabditis* *elegans* GABAergic neurons where mitochondrial dysfunction is induced by inhibiting respiration genetically or pharmacologically. In both mice and nematodes, reduction of autophagy restores axonal distribution of mitochondria, largely impaired when mitochondrial function is perturbed [12]. Thus, mouse RGCs expressing mutated Opa1 and nematode GABAergic neurons with dysfunctional mitochondria represent a useful tool to elucidate the retrograde signals linking mitochondrial dysfunction to mitophagy in neurons.

Alterations in Ca^2+^ signaling are a known consequence of mitochondrial dysfunction [13–15]. In normal conditions, mitochondria take up cytosolic Ca^2+^ via the mitochondrial uniport (MCU) using the driving force of the mitochondrial electrochemical gradient [16]. Mitochondrial dysfunction not only reduces the required driving force for Ca^2+^ uptake but it also triggers opening of the mitochondrial permeability transition pore, leading to the release of the Ca^2+^ accumulated in the matrix, further amplifying depolarization and reducing the Ca^2+^ buffering capacity of mitochondria [17]. The mitochondrial ability to take up Ca^2+^ is essential in cells such as neurons that are highly dependent on Ca^2+^ homeostasis. In neurons, mitochondria traffic to sites of high energy demand, such as Nodes of Ranvier, growth cones, and presynaptic endings, where they buffer Ca^2+^ and produce ATP to sustain neuronal communication and circuits formation [1, 18–20]. Moreover, synaptic mitochondria regulate Ca^2+^ fluctuations promoting ion gradient and, thereby, neurotransmission and synaptic plasticity [20, 21]. Therefore, altered cytosolic Ca^2+^ levels impair neurotransmission, axon guidance, and spine formation, leading to neuronal loss and organismic defects including cognitive dysfunction [15, 22]. Furthermore, sustained Ca^2+^ rises activate the Ca^2+^-dependent phosphatase calcineurin A (CnA) that dephosphorylates the master mitochondrial fission executor dynamin-related protein 1 (Drp1) to drive mitochondrial fragmentation, a prerequisite for mitophagy [23–25]. Active CnA also stimulates autophagy via TFEB dephosphorylation and nuclear translocation [26]. CnA is expressed in mature RGCs and amacrine cells but not in the other cells of the retina [27] and its overexpression promotes RGCs apoptosis [28], whereas the CnA inhibitor FK506 reduces RGC death in a mouse model of glaucoma [29].

In Opa1 deficient neurons, autophagosomes and fragmented mitochondria characterized by diminished Ca^2+^ buffering capacity accumulate in cell bodies [30–33]. Mechanistically, the diminished Ca^2+^ buffering capacity might not only result from the mitochondrial dysfunction associated with Opa1 deficiency but also from the dissociation of MICU1 from MCU that occurs when Opa1 is depleted [34]. Whether and how these facets of impaired mitochondrial function and Ca^2+^ buffering capacity are linked to the aggregation of autophagosomes observed in ADOA mouse models and to the AMPK-dependent mitophagy observed in RGCs expressing mutated Opa1 is unclear [35, 36].

With these questions in mind, we set out to investigate the role of Ca^2+^ in the activation of mitophagy observed in neurons where mitochondria are dysfunctional. We provide evidence that localized Ca^2+^ elevation at the axonal hillock activates mitophagy through the coordinated function of AMPK and CnA in both nematode and mouse neurons. Chelation of Ca^2+^ and inhibition of CnA normalizes autophagy, ultimately protecting neurons from mitochondrial dysfunction.

## Results

### Increased intracellular Ca^2+^ in nematode neurons and mouse RGCs with dysfunctional mitochondria

**Fig. 1:**
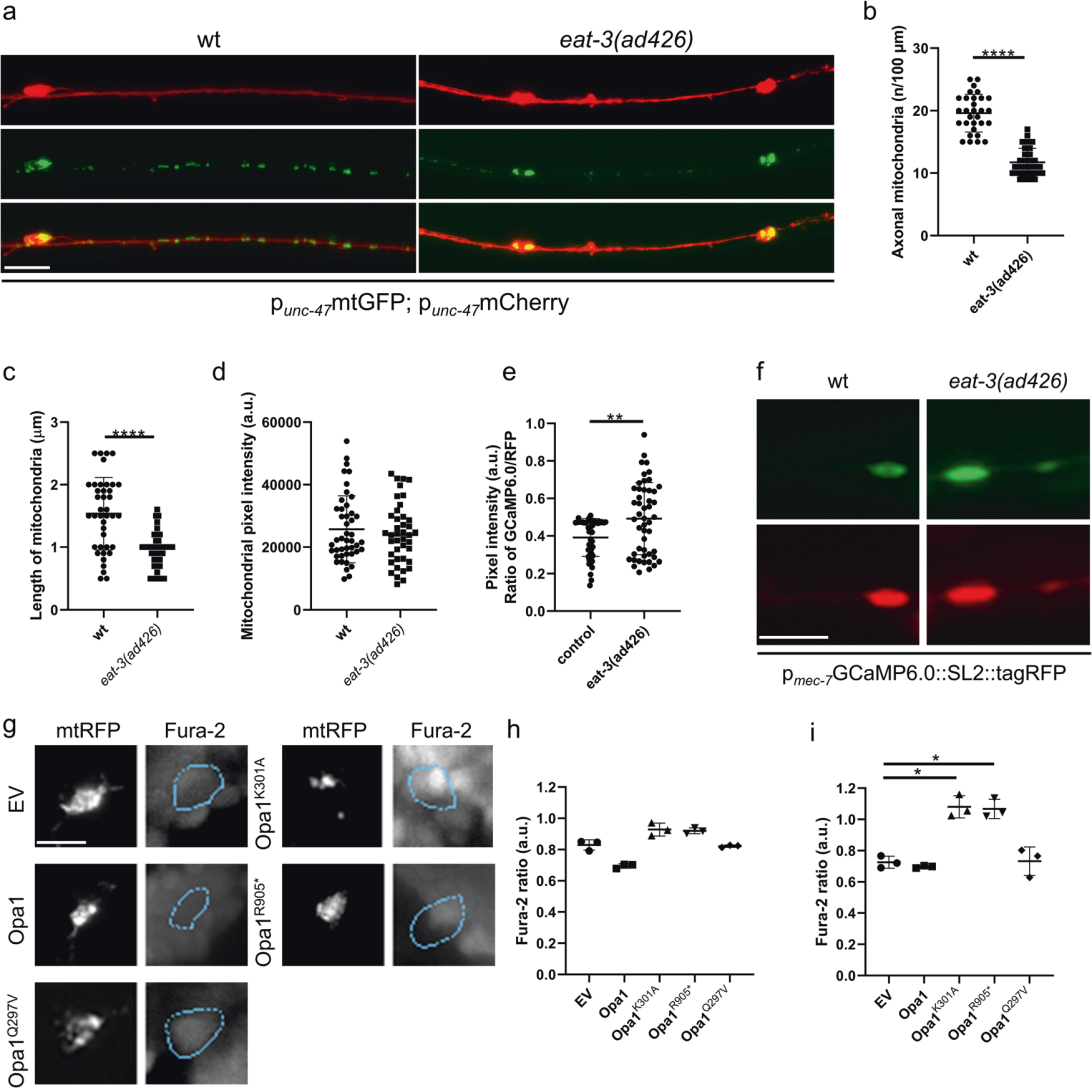
Opa1 mutations trigger Ca^2+^ elevation influencing autophagosomes and mitochondria distribution in both nematodes and RGCs. **a** Representative confocal images of wt and *eat-3(ad426)* nematodes coexpressing mtGFP and mCherry in GABAergic motor neurons. Top panels: axons (red); middle panels: mitochondria (green); bottom panels: merge. Scale bar, 20 μm. Quantification of mitochondrial axonal density (**b**), length (**c**), and mitochondrial pixel intensity (**d**) in experiments as in **a** (*n* = 25; NS, *P* > 0.05, ****P < 0.0001; Mann–Whitney test). **e** Representative images of the ratio between GCaMP6.0 and tagRFP in GABAergic motor neurons of wt and *eat-3(ad426)* nematodes. Scale bar, 20 μm. **f** Quantitative analysis of the GCaMP6.0/tagRFP ratio in experiments as in **c** (*n* = 35; ****P < 0.0001; Mann–Whitney test). **g** Representative images of Fura-2 ratio in RGCs transfected as indicated. Scale bar, 20 μm. **h**, **i** Quantitative analysis of Fura-2 ratio in soma (**f**) and axonal hillock (**g**) of RGCs cotransfected with mitochondria-targeted RFP and the indicated plasmids (*n* = 4 cells/experiment; *P < 0.033; one-way ANOVA with Dunnett’s T3 multiple comparison test). Data represent average ± SD of three independent experiments.

Reduced axonal mitochondrial density is a common feature of several neurodegenerative conditions associated with mitochondrial dysfunction and mitophagy. We decided to investigate the signals linking mitochondrial damage to mitophagy and reduced axonal mitochondrial density in a model of ADOA obtained by expressing pathogenic Opa1 mutants in RGCs and characterized by AMPK-dependent mitophagy and reduced axonal mitochondrial number [12]. In these cells, we expressed pathogenic Opa1 mutants (K301A and R905stop—R905*) as well as wild-type and constitutively active Q297V Opa1 as a control for Opa1 overexpression [12]. We also wished to generate a *C. elegans* model of mitochondrial damage in neurons caused by Opa1 disruption. To this end, we turned to nematode mutants of EAT-3, the homolog of mammalian Opa1. Similar to what observed in RGCs, mitochondrial density and length were reduced in axons of GABAergic neurons of *eat-3(ad426)* nematodes that lack EAT-3, whereas mitochondrial pixel intensity remained unaltered (Fig. 1a–d and Supplementary Fig. 1a). We then measured whether expression of the pathogenic Opa1 mutants affected mitochondrial function. These mutants did not reduce matricial ATP levels measured using the ratiometric ATP probe mtATeam in soma and neurite mitochondria (Supplementary Fig. 1b). However, fluorescence of the potentiometric dye tetramethylrhodamine methyl ester (TMRM) decayed in response to the ATP synthase inhibitor oligomycin [37], indicating that Opa1^K301A^ and Opa1^R905*^ mitochondria were latently dysfunctional (Supplementary Fig. 1c, d).

Mitochondrial dysfunction is a common cause of impaired mitochondrial Ca^2+^ buffering that together with low AMP:ATP ratio and reactive oxygen species (ROS) accumulation can activate AMPK [38–40]. Indeed, by real-time imaging, we measured increased cytosolic Ca^2+^ levels in both mechanosensory neurons of *eat-3(ad426)* mutant nematodes expressing the genetically encoded Ca^2+^ indicator GCaMP6.0 and in RGCs expressing pathogenic, but not wt or Q297V Opa1 mutants and stained with the ratiometric Ca^2+^ indicator Fura-2 (Fig. 1e–i). We noticed that the intensity of Fura-2 fluorescence was not uniformly higher throughout the soma of ADOA RGCs, but mostly increased at the level of the axonal hillock, proximal to mitochondria that we labeled by expressing a mitochondrially targeted RFP (mtRFP) (Fig. 1g). The signal GCaMP6.0 was instead uniform in the soma (Fig. 1f) even if mitochondria accumulated in the axonal hillock (Supplementary Fig. 1a). Conversely, Ca^2+^ increased in the axonal hillock when mitochondrial density and respiration was impaired by the redox cycler paraquat (Supplementary Fig. 2a, b). These results indicate that Opa1 mutation/depletion in the mouse and nematode neurons and pharmacologically induced mitochondrial dysfunction in *C. elegans* result in higher cytoplasmic Ca^2+^ levels.

### Ca^2+^ modulates axonal mitochondria density in ADOA neurons

We next addressed the relative role of mitochondrial depolarization and increased cytoplasmic Ca^2+^ levels in the observed phenotype of reduced axonal mitochondria density. Loss of mitochondrial membrane potential indeed triggers retrograde axonal transport of mitochondria [41], potentially explaining our observation. Conversely, high Ca^2+^ levels cause the dissociation of the mitochondrial adapter Miro from the Trak1/Milton complex to arrest mitochondrial movement and entry into axons. At a closer look, the few neurite mitochondria expressing Opa1^K301A^ and Opa1^R905*^ depolarized to a lesser extent than in the soma in response to oligomycin (Supplementary Fig. 1c, d). We wondered whether this was due to retrograde transport of dysfunctional organelles expressing pathogenic Opa1 mutants in the soma. We found that retrograde transport of mitochondria expressing pathogenic Opa1 mutants was not increased and instead these mitochondria were more stationary. Anterograde or retrograde velocity of the few motile Opa1^K301A^ and Opa1^R905*^ mitochondria was normal, suggesting that these mutants did not intrinsically alter mitochondrial transport speed (Supplementary Fig. 1e–h). Therefore, retrograde transport contributes at best marginally to the lower mitochondrial density in ADOA RGCs.

**Fig. 2:**
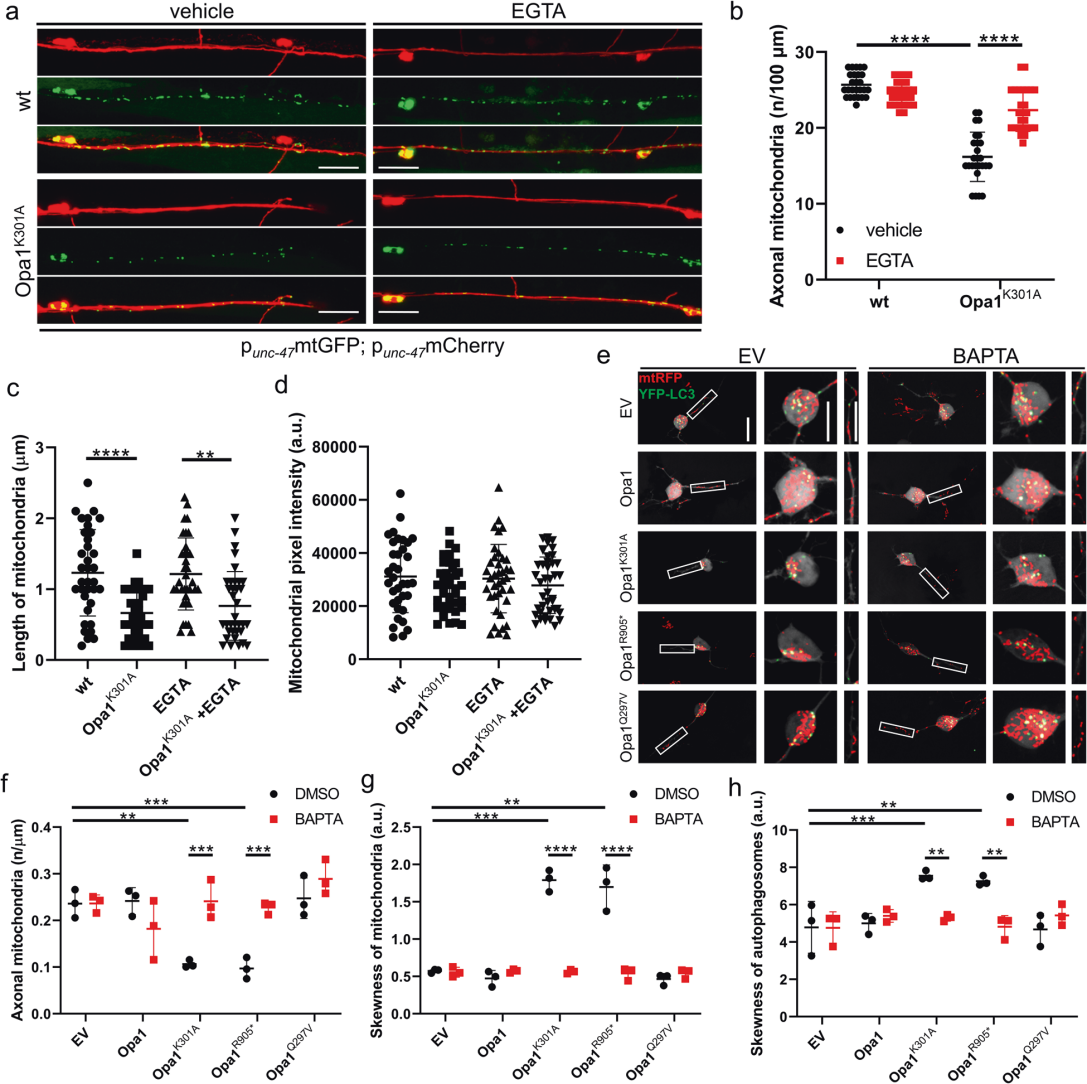
Chelation of Ca^2+^ restores autophagosomes and mitochondria distribution in neuronal axons. **a** Representative confocal images of transgenic wild-type and OPA1^K301A^ overexpressing nematodes coexpressing mtGFP and mCherry in GABAergic motor neurons. Top panels: axons (red); middle panels: mitochondria (green); bottom panels: merge. Where indicated, nematodes were treated with 10 mM EGTA. Scale bar, 20 μm. Quantification of mitochondrial axonal density (**b**), length (**c**), and mitochondrial pixel intensity (**d**) in experiments as in **a** (*n* = 26; ***P *<* 0.0001; two-way ANOVA with Sidak’s multicomparisons test). **e** Representative z-projections of stacks of confocal images of primary RGCs coexpressing mtRFP (red), YFP-LC3 (green), and the indicated plasmids. Where indicated, cells were treated with BAPTA. The cytoplasmic YFP-LC3 signal is pseudocolored in gray. Soma and boxed axonal regions are magnified in the right panels. The asterisk indicates the axon. Scale bars, 20 μm. **f** Mitochondrial content in axons of RGCs in experiments as in **c** (*n* = 9–19 cells/experiment; **P < 0.0021, ***P < 0.0002; two-way ANOVA with Sidak’s and Tukey’s multiple comparison tests). Quantification of mitochondria (**g**) and autophagosomes (**h**) accumulation in axonal hillocks (*n* = 10–22 cells/experiment; **P < 0.0021, ***P < 0.0002, ****P < 0.0001; two-way ANOVA with Sidak’s and Tukey’s multiple comparison tests). Data represent average ± SD of three independent experiments.

Next, we addressed whether elevated intracellular Ca^2+^ was responsible for the lower mitochondrial density in ADOA axons. To this end, we chelated intracellular Ca^2+^ and assessed the number of mitochondria in GABAergic motor neurons of nematodes expressing OPA1^K301A^ and in RGCs expressing the same mutant isoform. Supplementation of the chelating agents ethylene glycol tetraacetic acid (EGTA) to nematodes and 1,2-bis(2-aminophenoxy)ethane-*N,N,N*′*,N*′-tetraacetic acid (BAPTA) to RGCs restored axonal mitochondria density in OPA1^K301A^-expressing neurons (Fig. 2a–g). However, mitochondrial length and pixel intensity were not affected upon Ca^2+^ chelation (Fig.  2c, d). We repeated this experiment in nematodes treated with paraquat that also increases cytosolic Ca^2+^ levels and reduces mitochondrial content in *C. elegans* GABAergic neurons (Supplementary Fig. 2a–c). EGTA restored axonal mitochondria density in paraquat-treated nematodes (Supplementary Fig. 2d, e). Moreover, paraquat-induced mitophagy was abolished upon calcium chelation underlining the essential role of cytosolic Ca^2+^ elevation in the maintenance of mitochondrial homeostasis (Supplementary Fig. 2f, g). Altogether, these results indicate that the altered mitochondrial distribution observed in mouse and nematode models of neuronal mitochondrial dysfunction depends on intracellular Ca^2+^ elevation.

### CnA and AMPK inhibition restores axonal mitochondria density in ADOA neurons

In addition to its known role as an inhibitor of axonal mitochondrial transport, sustained Ca^2+^ levels could also regulate axonal mitochondrial content by activating mitophagy, via CnA-dependent stimulation of mitochondrial fission and AMPK activation. To verify if Ca^2+^-dependent mitophagy was responsible for the observed reduction in axonal mitochondrial content, we analyzed the effect of Ca^2+^ chelation on autophagy and on mitochondrial distribution. Consistent with our previous study, mitochondria and autophagosomes aggregated close to the axonal hillock in RGCs expressing pathogenic Opa1 mutants (Fig. 2c–f) [12]. Chelation of cytosolic Ca^2+^ restored autophagosomes and mitochondria distribution in the soma of RGCs (Fig. 2c–f), to an extent that was similar to that we observed upon autophagy inhibition [12]. Notably, Ca^2+^ chelation did not influence distribution of mitochondria and/or autophagosomes in Opa1 and Opa1^Q297V^-expressing RGCs, highlighting that treatment with the Ca^2+^ chelating agents does not damage healthy RGCs. These results place Ca^2+^ as an upstream second messenger in the induction of mitophagy in ADOA RGCs.

**Fig. 3:**
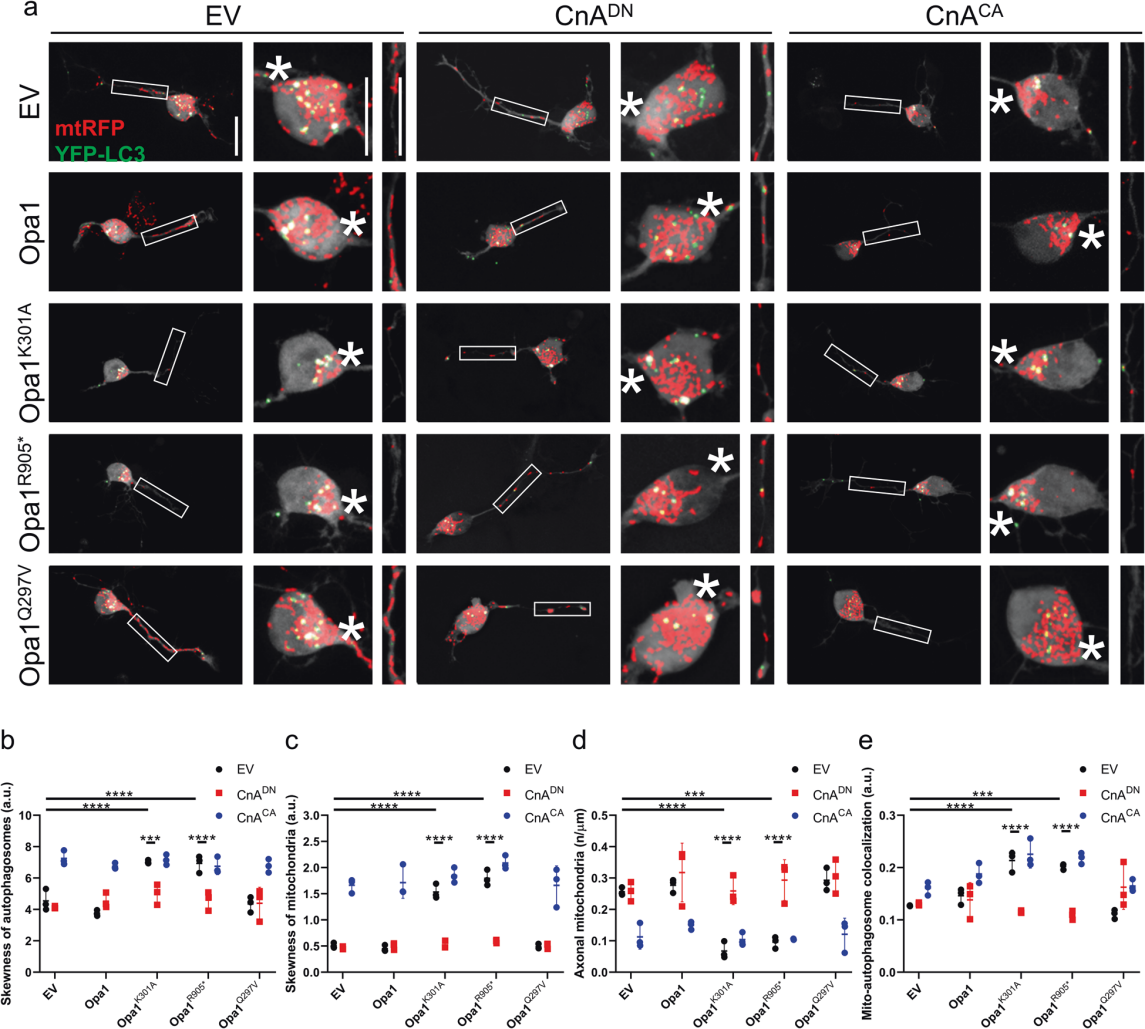
CnA inhibition restores mitochondrial distribution in axons of RGCs expressing pathogenic Opa1 mutants. **a** Representative z-projections of stacks of confocal images of RGCs coexpressing mtRFP (red), YFP-LC3 (green), and the indicated plasmids. The cytoplasmic YFP-LC3 signal is pseudocolored in gray. Soma and boxed axonal regions are magnified in the right panels. The asterisk indicates the axon. Scale bars, 20 μm. Quantification of autophagosomes (**b**) and mitochondria (**c**) accumulation in axonal hillocks from experiments as in **a** (*n* = 15–20 cells/experiment; ****P < 0.0001; two-way ANOVA with Sidak’s and Tukey’s multiple comparison tests). **d** Quantification of mitochondrial content in RGCs axons from experiments as in **a** (*n* = 16–28 cells/experiment; ***P < 0.0002, ****P < 0.0001; two-way ANOVA with Sidak’s and Tukey’s multiple comparison tests). **e** Quantification of autophagosome-mitochondria colocalization calculated using Manders’ coefficient in experiments as in **a** (*n* = 10–20 cells/experiment; ***P < 0.0002, ****P < 0.0001; two-way ANOVA with Sidak’s and Tukey’s multiple comparison tests). Data represent average ± SD of three independent experiments.

**Fig. 4:**
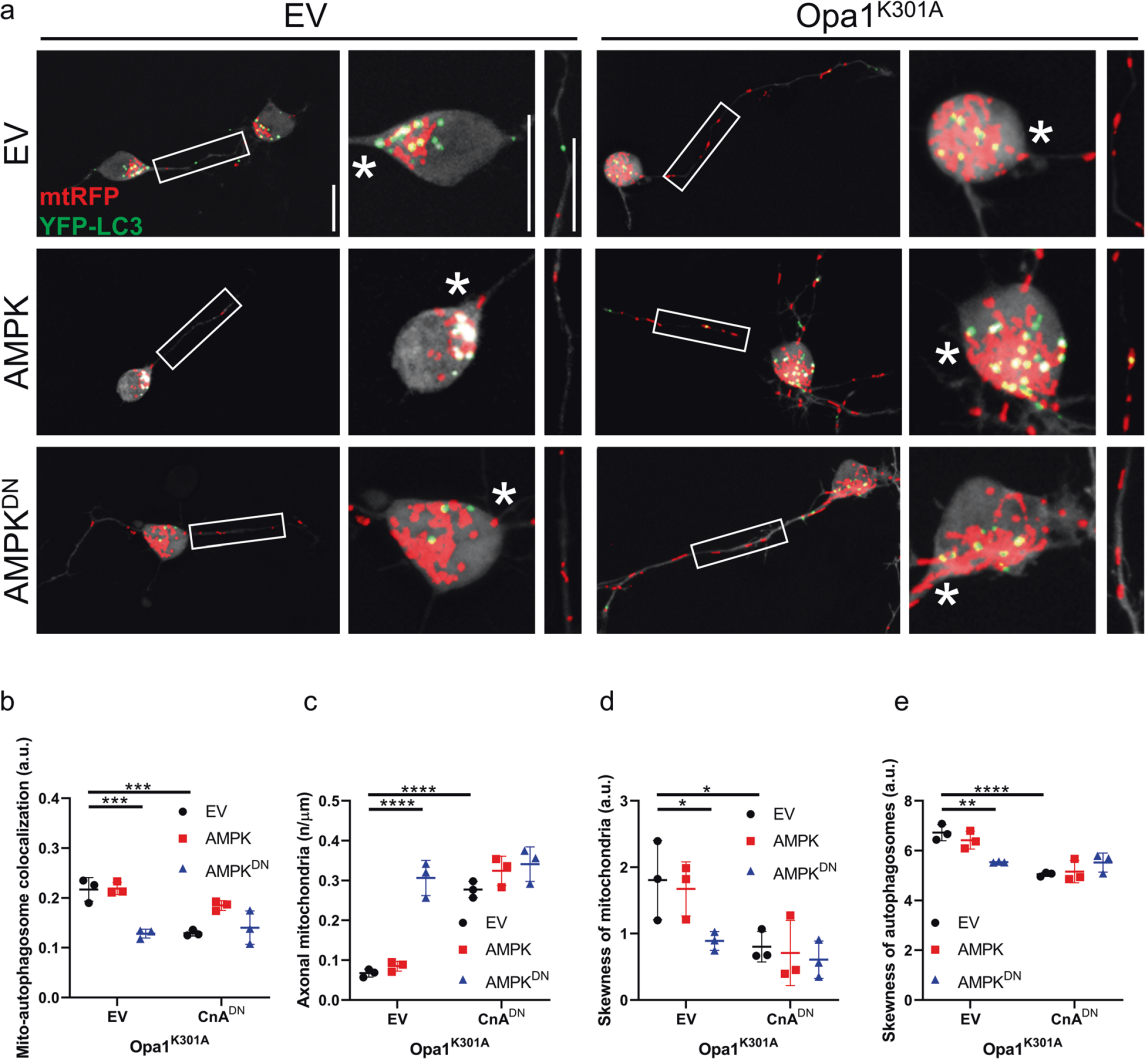
CnA and AMPK act in the same genetic pathway to regulate mitochondrial content in Opa1^K301A^ RGCs. **a** Representative z-projections of stacks of confocal images of primary RGCs coexpressing mtRFP (red), YFP-LC3 (green), and the indicated plasmids. The cytoplasmic YFP-LC3 signal is pseudocolored in grey. Soma and boxed axonal regions are magnified in the right panels. The asterisk indicates the axon. Scale bars, 20 μm. **b** Quantification of autophagosome-mitochondria colocalization calculated using Manders’ coefficient in experiments as in **a** (*n* = 16–20 cells/experiment; ***P < 0.0002; two-way ANOVA with Sidak’s and Tukey’s multiple comparison tests). **c** Quantification of axonal mitochondria content in experiments as in **a** (*n* = 14–20 cells/experiment; ****P < 0.0001; two-way ANOVA with Sidak’s and Tukey’s multiple comparison tests). Quantification of autophagosomes (**d**) and mitochondria (**e**) accumulation in axonal hillocks in experiments as in **a** (*n* = 15–20 cells/experiment; *P < 0.033, **P < 0.0021, ****P < 0.0001; two-way ANOVA with Sidak’s and Tukey’s multiple comparison tests).

We next investigated the role of the Ca^2+^-dependent phosphatase CnA in mitochondrial distribution and mitophagy in ADOA RGCs by expressing a constitutively active (CnA^CA^) and a dominant negative (CnA^DN^) mutant of CnA [12]. Interestingly, inhibition of CnA restored the distribution of mitochondria in the soma and the mitochondrial density in axons of ADOA RGCs (Fig. 3a–d). While CnA^DN^ did not influence mitochondrial or autophagosomal distribution in Opa1 and Opa1^Q297V^-expressing RGCs, CnA^CA^ expression was sufficient to trigger the accumulation of mitochondria and autophagosomes at the axonal hillock and depletion of axonal mitochondria (Fig. 3a–d). Furthermore, colocalization of autophagosomes and mitochondria in RGCs appeared to correlate with CnA activity, as it decreased upon CnA^DN^ and increased upon CnA^CA^ expression (Fig. 3a, e). Notably, CnA^DN^ did not affect autophagic flux in RGCs expressing pathogenic Opa1 mutants, indicating that the observed effects of CnA on mitochondrial density were not a consequence of changes in basal autophagy levels (Supplementary Fig. 3a, b). Because CnA can dephosphorylate and deactivate AMPK, we next addressed whether CnA and AMPK acted in the same genetic pathway to regulate mitochondrial content and autophagosomes formation in ADOA RGCs. Simultaneous genetic inhibition of both CnA and AMPK in Opa1^K301A^-expressing RGCs did not result in any additive effect on mitophagy (Fig. 4a, b), density of axonal mitochondria (Fig. 4a, c), and distribution of autophagosomes and mitochondria (Fig. 4a, d, e). Therefore, we conclude that CnA and AMPK are in the same pathway controlling axonal hillock accumulation of autophagosomes and mitochondria in ADOA RGCs.

### Neuronal cell death caused by mitochondrial dysfunction is inhibited by Ca^2+^ chelation and CnA blockage

**Fig. 5:**
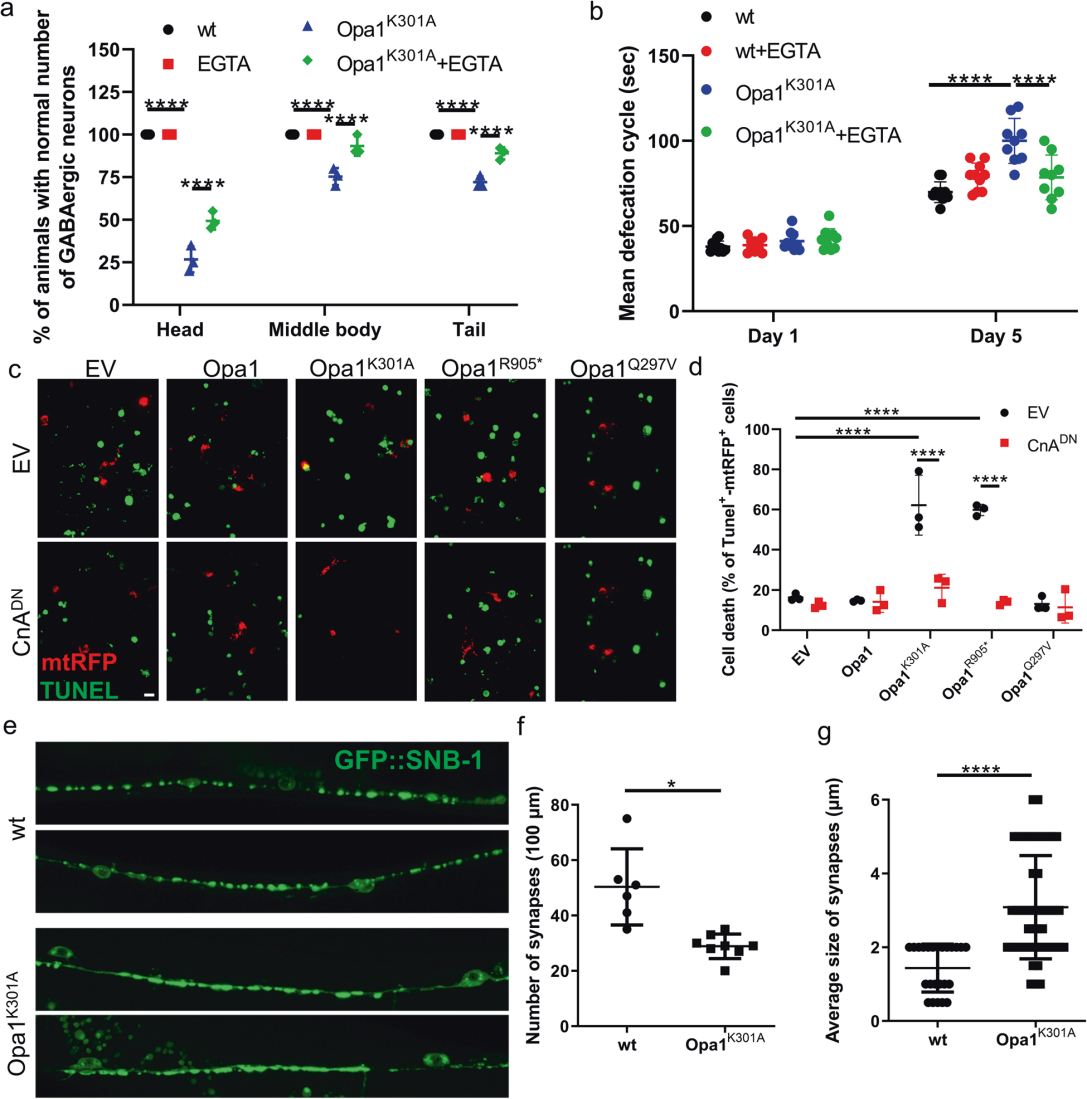
Ca^2+^/CnA blockage inhibits OPA1^K301A^ and OPA1^R905*^-triggered apoptosis in RGCs and neuronal defects in nematodes. **a** Quantification of Opa1^K301A^-expressing GABAergic motor neurons death in nematodes treated as indicated (*n* = 30; ****P < 0.0001; two-way ANOVA with Tukey’s multiple comparison test). **b** Quantification of defecation motor program in transgenic nematodes expressing OPA1^K301A^ treated with EGTA where indicated (*n* = 40 animals; ****P < 0.0001; two-way ANOVA with Tukey’s multiple comparison test). **c**, **d** Quantification of apoptosis in RGCs transfected with the indicated plasmids (*n* = 40 cells/experiment; *P < 0.0332; ****P < 0.0001; two-way ANOVA with Sidak’s multiple comparison test). **e** Representative confocal images of the fluorescence of GFP::SNB-1 in transgenic animals expressing Opa1^K301A^. Bar, 20 μm. SNB-1 synaptobrevin. Number (**f**) and size (**g**) of synapses in experiments as in **e**. Data represent average ± SD of 24–49 animals. *P < 0.0332; ****P < 0.0001; unpaired *t*-test with Welch’s correction (**c**) and Mann–Whitney test (**d**).

We next addressed whether the Ca^2+^/CnA pathway identified participated in neuronal death caused by mitochondrial dysfunction. We first confirmed that expression of pathogenic Opa1 mutants triggers cell death also in nematodes (Fig. 5a, Supplementary Fig. 4), as it does in RGCs (Fig. 5c, d and [12]). To investigate the contribution of elevated intracellular Ca^2+^ in Opa1^K301A^-mediated cell death, we examined the survival of *C. elegans* GABAergic motor neurons upon Ca^2+^ chelation. EGTA protected against Opa^K301A^-driven neuronal death (Fig. 5a). The GABAergic neuronal circuit in *C. elegans* consists of 26 neurons, which regulate locomotion, foraging, and defecation behavior [42, 43]. To extend our analysis, we monitored the defecation cycle of Opa1^K301A^-expressing nematodes and evaluated the neuronal function of GABAergic neurons in response to Ca^2+^ chelation. Supplementation with EGTA restored the defecation cycle of Opa1^K301A^-expressing animals (Fig. 5b), suggesting that Ca^2+^ chelation not only protects neurons from death but also spares their function. Defecation is mainly regulated by AVL and DVB GABAergic motor neurons, which are localized in the head and tail region respectively [43]. These two neuronal cells are more susceptible to the deleterious effect of Opa1^K301A^ compared to other GABAergic neurons (Supplementary Fig. 4a). These results can be explained either by the mosaicism expression of Opa1^K301A^ in GABAergic neurons or by the specific anatomical features of the respective neurons, such as axon morphology, cell body placement, and synaptic connectivity. To examine the synaptic morphology of GABAergic motor neurons, we used the synaptic vesicle marker SNB-1/synaptobrevin fused with GFP. Opa1^K301A^-expressing nematodes display fewer SNB-1::GFP puncta and larger synaptic regions in GABAergic motor neurons (Fig. 5e–g), a finding consistent with the observed reduction in synapses in mammalian cortical neurons where Opa1 was ablated [30] and in RGCs and hippocampal neurons of an ADOA mouse model [44, 45].

Several mitochondrial mutations have been associated with excess ROS accumulation leading to oxidative stress and neurodegeneration [2, 46–49]. To investigate the contribution of ROS in our model, we treated OPA1^K301A^-expressing nematodes with the antioxidant N-acetylcysteine (NAC). Interestingly, NAC protects against neurodegeneration of GABAergic motor neurons indicating that the expression of OPA1^K301A^ results in increased ROS generation and neuronal loss (Supplementary Fig. 4b). Simultaneous supplementation of NAC and EGTA did not display any additive neuroprotection, highlighting that altered ROS and calcium levels act in parallel to mediate neuronal dysfunction and loss in OPA1^K301A^-expressing nematodes (Supplementary Fig. 4b). Comforted by the remarkable conservation of the pathway, we examined the impact of CnA in Opa1^K301A^-mediated apoptosis in RGCs and found that genetic inhibition of CnA spared RGCs from death (Fig. 5c, d), similarly upon autophagy inhibition [12] (Supplementary Fig. 4c). Our findings in their totality underline Ca^2+^ signaling as a central denominator of neuronal loss in ADOA pathogenesis.

## Discussion

**Fig. 6:**
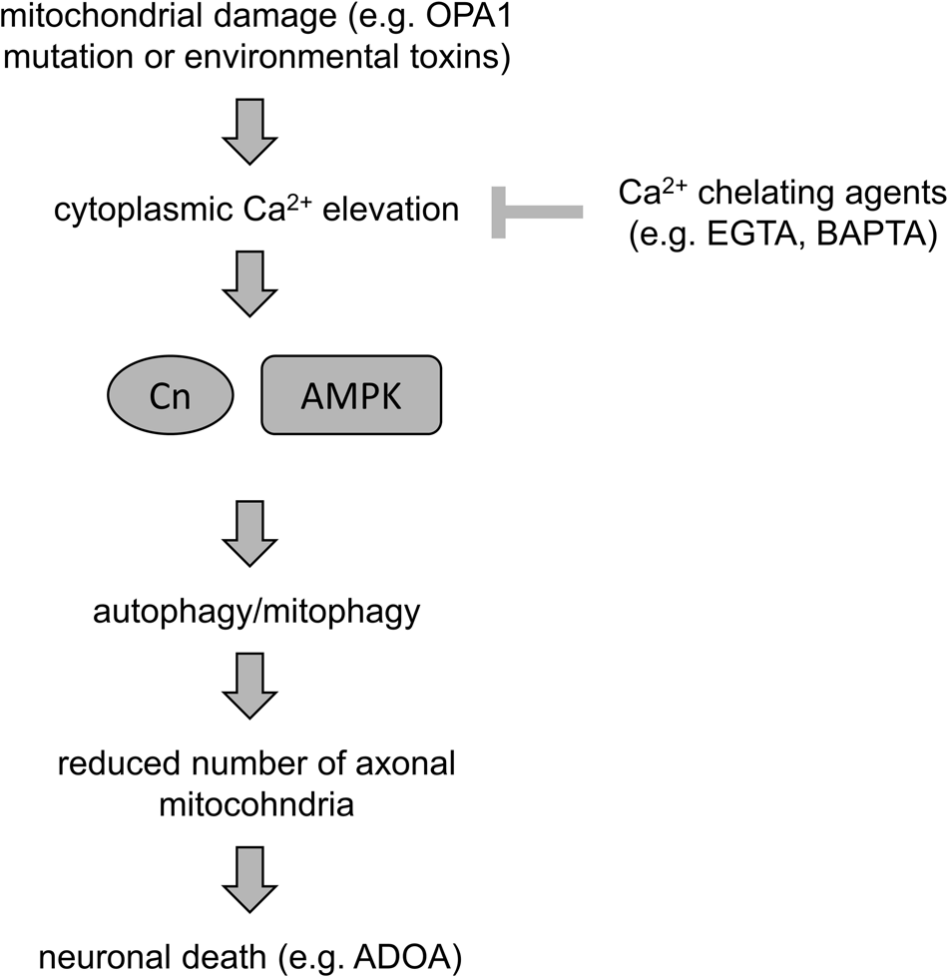
CnA–AMPK-mediated mitophagy depletes axonal mitochondria and triggers neurodegeneration. Perturbation of mitochondrial homeostasis, which is induced either by environmental toxins (e.g., paraquat) or genetic mutations (e.g., OPA1^K301A^), results in elevation of cytoplasmic Ca^2+^ levels. In turn, a Ca^2+^-dependent signaling cascade, which involves Cn and AMPK stimulation, mediates mitophagy to eliminate damaged organelles and sustain cellular viability. Chronic mild mitochondrial dysfunction and/or long-term exposure to environmental stressors could promote runaway mitophagy, which subsequently reduces mitochondrial population, triggers energetic crisis, and eventually neuronal cell death. Modulation of mitophagy levels increases mitochondrial load in neuronal processes preventing neurodegeneration.

The precise signals linking mitochondrial dysfunction and dysmorphology to mitophagy are unclear, especially in neurons where mitophagy is a recognized mechanism of neurodegeneration. Here, we demonstrate that in two types of neurons, RGCs and GABAergic motor neurons, from two different model organisms, *Mus musculus* and *C. elegans*, mutation or deletion of the key mitochondrial protein Opa1 causes uncontrolled mitophagy by recruiting a CnA–AMPK pathway activated by elevated intracellular Ca^2+^ levels (Fig. 6).

Impaired mitochondrial function and mitophagy are common features of several neurodegenerative diseases [2, 50]. Although mitophagy is usually associated with neuroprotection, also uncontrolled mitochondrial degradation could reduce mitochondrial density, overwhelming the reserve capacity of the remaining organelles and ultimately leading to bioenergetic crisis and neuronal loss [51, 52]. In fact, excessive mitophagy is pathogenetic in ADOA, where AMPK activation and ULK1 recruitment at axonal hillocks leads to localized autophagosome accumulation and mitophagy in RGCs expressing mutated Opa1 [12]. This mechanism appears genetically conserved in *C. elegans* GABAergic motor neurons. Because the connection between Opa1 inhibition and activation of mitophagy was conserved across species and neuronal types, we deemed this model suitable to investigate the signals linking mitochondrial dysfunction to the activation of AMPK-dependent mitophagy and diminished mitochondrial density in RGCs axons. Local Ca^2+^ signals are crucial in neuronal function and in controlling mitochondrial transport along axons. Our data indicate that Ca^2+^ also participates in mitochondrial degradation in *C. elegans* GABAergic motor neurons and mouse RGCs. The conservation of this mechanism in mammals and nematodes may suggest its conservation in other neuronal types and species. Our data demonstrate that the local increase of Ca^2+^ engages AMPK to drive mitophagy close to the axonal hillocks of neurons expressing pathogenic Opa1 mutants. This process ultimately determines the content of mitochondria in axons and the functioning and survival of neurons. Mechanistically, increased intracellular Ca^2+^ levels can activate AMPK via CAMKKbeta, directly linking this universal second messenger to mitophagy.

Our results indicate a role for CnA in the pathway leading to mitophagy in ADOA RGCs. An epistatic analysis places CnA and AMPK in the same pathway controlling axonal mitochondrial density but does not allow to understand whether CnA is placed up or downstream AMPK directly in the control of mitophagy. CnA can influence autophagy and mitophagy in multiple ways. First, CnA can dephosphorylate the crucial autophagy transcription factor TFEB, driving its nuclear relocalization and lysosome/autophagy genes transcription [26]. However, in RGCs genetic activation or blockage of CnA does not alter autophagic flux, suggesting that in this model CnA does not directly modulate TFEB and autophagy. Second, CnA can dephosphorylate and deactivate AMPK [53]. However, mitophagy in both RGCs and GABAergic neurons is curtailed by inhibition, not activation of CnA. These results suggest that CnA participates at a different checkpoint for mitophagy. Indeed, CnA also controls Drp1 dependent mitochondrial fission, a prerequisite for mitochondrial engulfment by autophagosomes [23, 25]. Altogether, we therefore interpret the beneficial effect of CnA inhibition on excess mitophagy as a consequence of Drp1 inhibition. In other systems, Drp1 inhibition can indeed counteract the deleterious effects of Opa1 deletion, lending further support to this hypothesis [54]. Drp1 is tightly linked with mitochondrial life cycle, since its activity could promote mitochondrial fission leading to the generation of new mitochondria and also facilitate the removal of defective organelles via mitophagy induction [55, 56]. Thus, Drp1 inhibition could also shift the balance of mitochondrial dynamics toward fusion, increasing the health of mitochondria and improving neuronal survival in pathological conditions, where excessive fission and mitophagy occur. Our results also suggest that drugs such as the CnA inhibitor FK506 might interfere with the natural history of ADOA, in addition to, or instead of gene therapy [57].

Our results add autophagy as a mechanism controlling mitochondrial subcellular distribution in polarized cells such as mouse RGCs and *C. elegans* neurons. Whether our findings can be extended to other cell types, organelles and disease models remain to be explored. However, it shall be noted that clustering and abnormal distribution of mitochondria has been reported in models of amyotrophic lateral sclerosis, Alzheimer’s, Parkinson’s, and Huntington’s disease [3, 19, 20, 58–60]. Our findings provide insights into the molecular mechanism of ADOA pathogenesis and can stimulate a deeper understanding of the contribution of mitophagy to neuronal pathophysiology and survival.

## Materials and methods

### Strains and genetics

We followed standard procedures for *C. elegans* strain maintenance [1]. Nematode rearing temperature was kept at 20 °C. The following strains were used in this study: N2: wild-type Bristol isolate and DA631: *eat-3(ad426)II*; *him-8(e1489)IV*. To monitor mitochondrial number and distribution in neurons, we used EG6531: N2;*oxIs608*[p_*unc-47*_mCherry];*oxEx1182*[p_*unc-47*_TOM-20::GFP] [2], IR4554: *eat-3(ad426)II*; *him-8(e1489)IV*;*oxIs608*[p_*unc-47*_mCherry];*oxEx1182*[p_*unc-47*_TOM-20::GFP], and IR2097: N2;*oxIs608*[p_*unc-47*_mCherry];*oxEx1182*[p_*unc-47*_TOM-20::GFP];*Ex0023*[p_*unc-47*_OPA1^K301A^] [12]. To examine neuronal cytoplasmic calcium levels in basal and oxidative stress conditions, we used IR1139: N2;*Ex002*[p_*mec-17*_GCaMP2.0] and AQ3236: *IjSi2[*p_*mec-7*_*GCaMP6m::SL2::tagRFP; unc-119(+)]II; unc-119(ed3)III*. To investigate defecation motor program, we used IR2093: N2;*Ex0023*[p_*unc-47*_OPA1^K301A^] transgenic animals [12].

### Plasmids and molecular biology

peYFP-hLC3 (YFP-LC3), mito-dsRED (mtRFP), pDCR-HA-ΔCnA (CA), pDCR-HA-ΔCnA^H151Q^ (DN), pDCR-CnB, pCMV-AMPK (AMPK), pCMV-AMPK^T172A^ (DN), pMSCV, pMSCV-Opa1, pMSCV-Opa1^K301A^ pMSCV-Opa1^Q297^, and pMSCV-Opa1^R905*^ were previously described [12].

### RGC purification and culture

All mice procedures were performed according to approved protocols (protocol 32/2011 CEASA and 318/2015 Ministry of Health to LS). RGCs were purified from 8 to 10 C57Bl/6J aged P0–P2 using the immuno-panning magnetic protocol as previously described [61]. Overall, 1 × 10^4^ cells were transfected with the indicated plasmids using Neon Transfection System (Invitrogen) and seeded onto 24-mm round glass coverslips precoated with poly-L-ornithine 0.2 mg/ml (Sigma) and laminin 0.5 mg/ml (Roche). Cells were cultured as described at 37 °C in a 5% CO_2_ atmosphere [61]. All experiments were performed 24 h after seeding. Autophagy was inhibited with 200 nM bafilomycin A (Sigma) for 30 min or with 10 mM 3-Methyladenine (Sigma) for 24 h. Ca^2+^ was chelated with 40 µM BAPTA-AM (Sigma) for 30 min.

### Assessment of cytoplasmic Ca^2+^ levels

For intracellular Ca^2+^ assessment in nematodes, transgenic animals expressing the Ca^2+^reporter GCAMP2.0 or GCaMP6.0 fused with RFP in six touch receptor neurons were examined under a Zeiss AxioImager Z2 epifluorescence microscope. Four-day-old adult hermaphrodites were exposed to paraquat at a final concentration of 10 mM. Animals were imaged after 2 days at 20 °C. Worms were immobilized with levamisole before mounting on 2% agarose pads for microscopic examination with a Zeiss AxioImager Z2 epifluorescence microscope. Images were acquired under the same exposure. Average pixel intensity values were calculated by sampling images of different animals. We calculated the mean pixel intensity of GCAMP2.0 or both GCaMP6.0 and RFP for each neuron in these images using the ImageJ software (http://rsb.info.nih.gov/ij/). GCaMP6.0 mean values were normalized to RFP mean values and then compared using unpaired *t*-tests. For each experiment, at least 35 animals were examined for each condition. Each assay was repeated at least three times. We used the Prism software package (GraphPad Software) for statistical analyses.

For Ca^2+^ imaging in RGCs, cells were loaded with 1 µM Fura2-AM (Invitrogen) dissolved in culture media for 20 min at 37 °C. Cells were washed in Ringer’s solution (Sigma) and then placed on the stage of an Olympus IMT-2 inverted microscope (Melville, NY) equipped with a CellR imaging system. Images of the 340 and 380 nm fluorescence emission from mtRPF-expressing RGCs were acquired upon excitation at 510 nm and then processed using the Multi Measure plugin of ImageJ following background subtraction and expressed as Fura2-AM 340/380 ratio.

### Mitochondrial imaging

Four-day-old adult hermaphrodites coexpressing mitochondria-targeted GFP together with cytoplasmic mCherry in GABAergic motor neurons were used in our study. Animals were immobilized with levamisole before mounting on 2% agarose pads for microscopic examination with Zeiss AxioObserver Z1 confocal microscope. Imaging parameters such as microscope and camera settings (lens and magnifier used, filters exposure time, resolution, laser intensity, gain, etc.) were kept the same during the imaging process. The quantification of mitochondrial number was performed using Zeiss ZEN 2012 software to automatically threshold the images and then determine the outlines of GFP-targeted mitochondria in axons of GABAergic motor neurons. Mitochondrial number was calculated by counting the average number of puncta per 100 μm of axonal length.

For confocal imaging in RGCs, images were acquired using a laser scanning microscope (TCS SP5, Leica) equipped with the LasAF software (Leica). YFP and dsRED were excited using the 488 nm or the 543 nm line of the HeNe and Argon with a 63×, 1.4NA objective. Twenty confocal images were acquired along the *z*-axis, deconvolved, and 3D reconstructed using ImageJ. Autophagosomes and mitochondria colocalization was quantified using Manders’ coefficient [45]. The length of axons was manually traced and quantified using the Multi Measure plugin of ImageJ. The distribution of mitochondria and autophagosomes in the soma was quantified using the Skewness coefficient of ImageJ.

For mitochondrial transport imaging, 1 × 10^4^ cells seeded onto 24-mm round glass coverslips were transfected with mtRFP and with the indicated plasmids. After 24 h, cells were placed on a thermostated chamber at 37 °C and maintained on the stage of an Olympus inverted microscope equipped with a CellR imaging system. Sequential images of the 584 nm fluorescence emission were acquired every 1 s with a 60×, 1.4NA objective (Olympus) using the CellR software and then processed using the Straighten plugin of ImageJ. Mitochondrial velocity was measured as described [62].

For evaluation of membrane potential, 1 × 10^4^ RGCs were plated on 24 mm round coverslips and were cotransfected with pEGFP and the indicated plasmids. After 24 h, RGCs were loaded with 0.5 nM TMRM (Sigma) dissolved in Hanks’ balanced salt solution (HBSS, Invitrogen) supplemented with 10 mM HEPES pH 7.4 (Invitrogen) in the presence of 2 mg/ml cyclosporine H (a P-glycoprotein inhibitor, Sigma) for 30 min at 37 °C. Cells were then placed on the stage of an Olympus IMT-2 inverted microscope (Melville, NY) equipped with a CellR imaging system. Cells were excited using a 525/20 BP excitation filter, and emitted light was acquired using a 570/LP filter. Imaging and analysis of TMRM fluorescence over mitochondrial regions of interest was performed as described [63].

Mitochondrial ATP content was determined by FRET image analysis of neurons seeded onto 24-mm round glass coverslips after electroporation with the indicated plasmid and ATeam as described [64]. Basal values are average of 3 min resting fluorescence ratios recorded after cells were allowed to equilibrate for 5 min in the indicated HBSS buffers.

### Analysis of apoptosis

RGCs were transfected using mtRFP and Opa1 plasmids. After 24 h, cells were fixed for 10 min at room temperature with 3.7% (w/v) formaldehyde and permeabilized for 10 min with Triton-X-100 0.1%. Apoptosis was evaluated by TUNEL using the In-Situ Cell Death Detection Kit (Roche) following manufacturer’s instructions. Images were acquired as single planes using a Nikon Eclipse TE300 inverted microscope equipped with a spinning-disk Perkin-Elmer Ultraview LCI confocal system. dsRED and TUNEL-FITC were excited using the 568 nm or the 440 nm line of the HeNe laser (Perkin-Elmer) using a 63×, 1.4NA objective.

### Statistical analysis

We used the Prism 8.3 software package (GraphPad Software) for statistical analyses. In graphs, average ± SD is plotted, and *P* values are indicated in figure legends. *T*-test and one-way or two-way ANOVA tests were used to determine statistical significance. The indicated corrections were used if assumptions of normality and homoscedasticity were not respected. *P* < 0.05 was considered statistically significant.

## Supplementary information

Supplementary online material
