# Supplementary material for: Sustained intracellular calcium rise mediates neuronal mitophagy in models of autosomal dominant optic atrophy
Source: Cell Death Differ. 2021 Aug 13;29(1):167–77. doi: 10.1038/s41418-021-00847-3 (PMC8738763; doi:10.1038/s41418-021-00847-3)
Supplement: Supplementary file 1 — Supplementary online material [file 41418_2021_847_MOESM1_ESM.docx]

**Sustained intracellular calcium rise mediate neuronal mitophagy in models of autosomal dominant optic atrophy.**

Marta Zaninello, Konstantinos Palikaras et al

**Supplementary online figures**

**
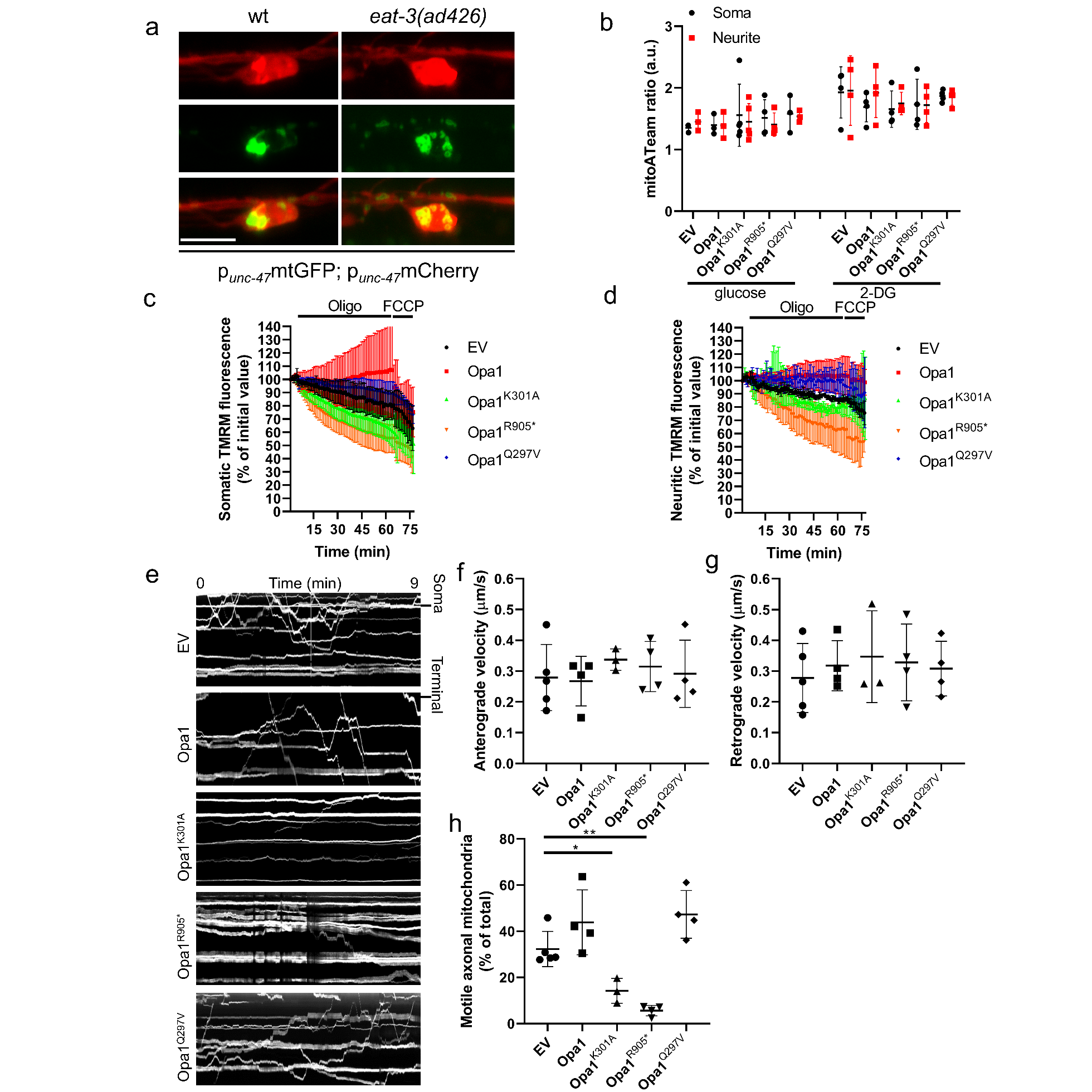
**

**Supplementary Figure 1. Opa1 modulates mitochondrial distribution, function, and motility.**

**(a)** Representative images of *eat-3(ad426)* GABAergic cell bodies. The channels of neurons (red, top) mitochondria (green, middle) and merged (bottom) are shown. Scale bar, 20 μm.

**(b)** Mitochondrial ATP levels in soma and neurites (n=1-5 cells/experiment; n.s. in a one-way ANOVA test with Tukey’s and Sidak’s multiple comparison tests).

**(c, d)** Average TMRM fluorescence changes over somatic (c) and neurite (d) mitochondria of GFP^+^ RGCs co-transfected with GFP and the indicated plasmids (n=2-8 cells/experiment of 3-6 independent experiments). Where indicated, cells were treated with Oligomycin (Oligo, 2 µM) and FCCP (2 µM).

**(e)** Representative kymographs of movies of the fluorescence of mtRFP acquired 24 hrs after transfection of primary RGCs co-transfected with mtRFP and the indicated plasmids.

**(f, g)** Anterograde (f) and retrograde (g) velocity of mitochondria (n=1-5 cells/experiment; n.s. p<0.0021 in a one-way ANOVA test with Dunnett’s multiple comparison test).

**(h)** Mitochondrial movement in neurites. The number of moving mitochondria is normalized to the number of total mitochondria (n=1-5 cells/experiment; *, p<0.03; **, p<0.002 in a one-way ANOVA test with Dunnett’s multiple comparison test).

**
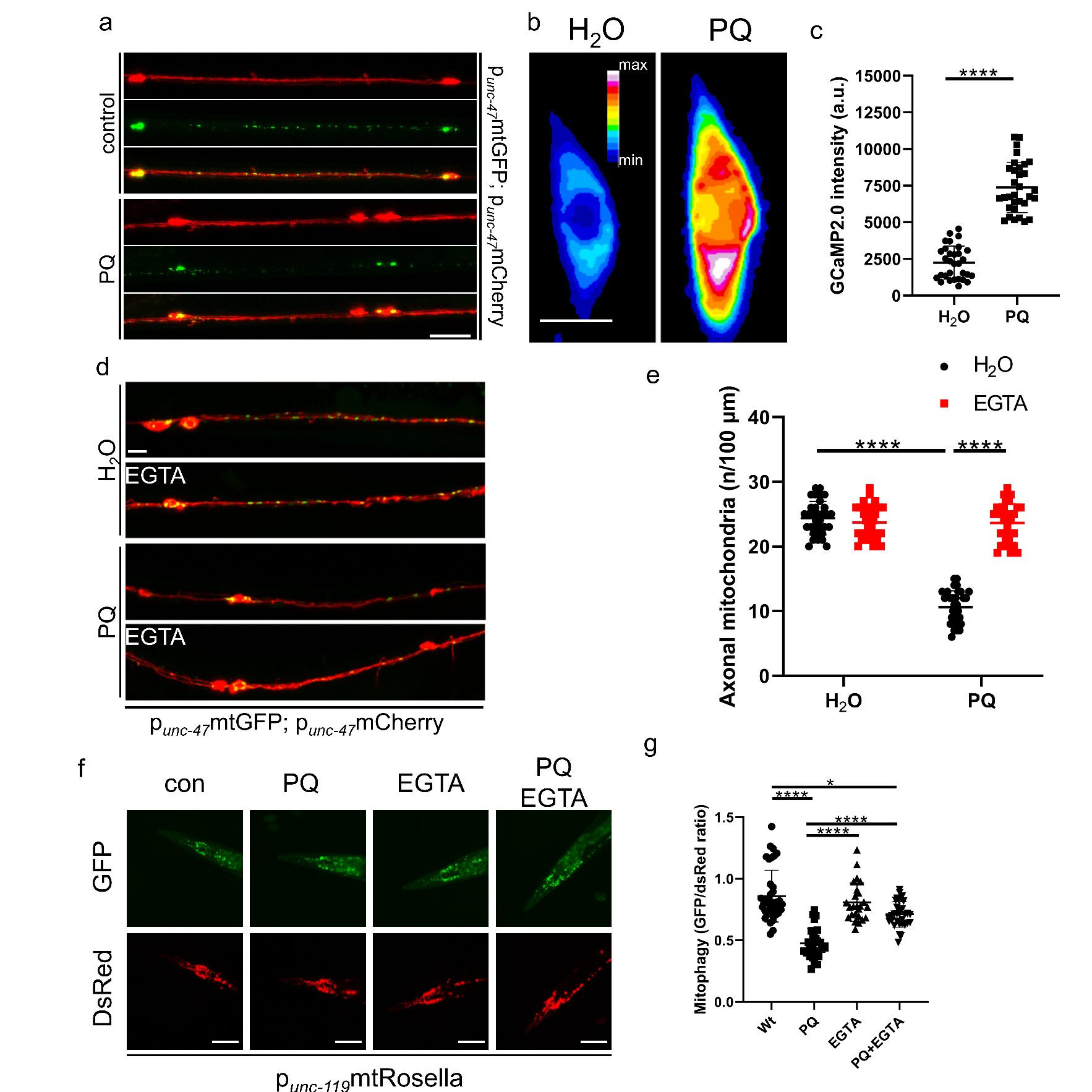
**

**Supplementary Figure 2. Cytoplasmic Ca^2+^ levels mediate mitochondrial distribution in axons of paraquat-treated nematodes.**

**(a)** Representative images of GABAergic motor neurons in nematodes of the indicated genotype treated where indicated with paraquat (PQ). The channels of neurons (mCherry, red, top) mitochondria (mtGFP, green, middle) and merged (bottom) are shown. Scale bar, 20 μm.

**(b)** Representative pseudocolored images of GCaMP2.0 fluorescence in mechanosensory nematode neurons treated as indicated. Scale bar, 20µm.

**(c)** Average±SD of GCaMP2.0 fluorescence in experiments as in b (n=35; *****P*<0.0001; unpaired *t-*test with Welch’s correction).

**(d)** Experiments were as in a except that where indicated, nematodes were treated with 10mM EGTA.

**(e)** Average±SD of axonal mitochondrial content in experiments as in d (n=25; *****P*<0.0001; unpaired *t-*test with Welch’s correction).

**(f)** Representative images of the GFP and dsRed signals in neuronal cells of transgenic animals expressing the mitophagy sensor mtRosella treated with PQ and 10 mM EGTA where indicated. A decreased ratio between pH-sensitive GFP and pH-insensitive DsRed indicates mitophagy induction. Scale bars, 20 μm

(g) Average±SD of GFP/dsRed ratio in experiments as in f. (n=45 animals/experiment). *, p<0.03; *****P*<0.0001; two-way ANOVA with Sidak’s multiple comparison test.

**
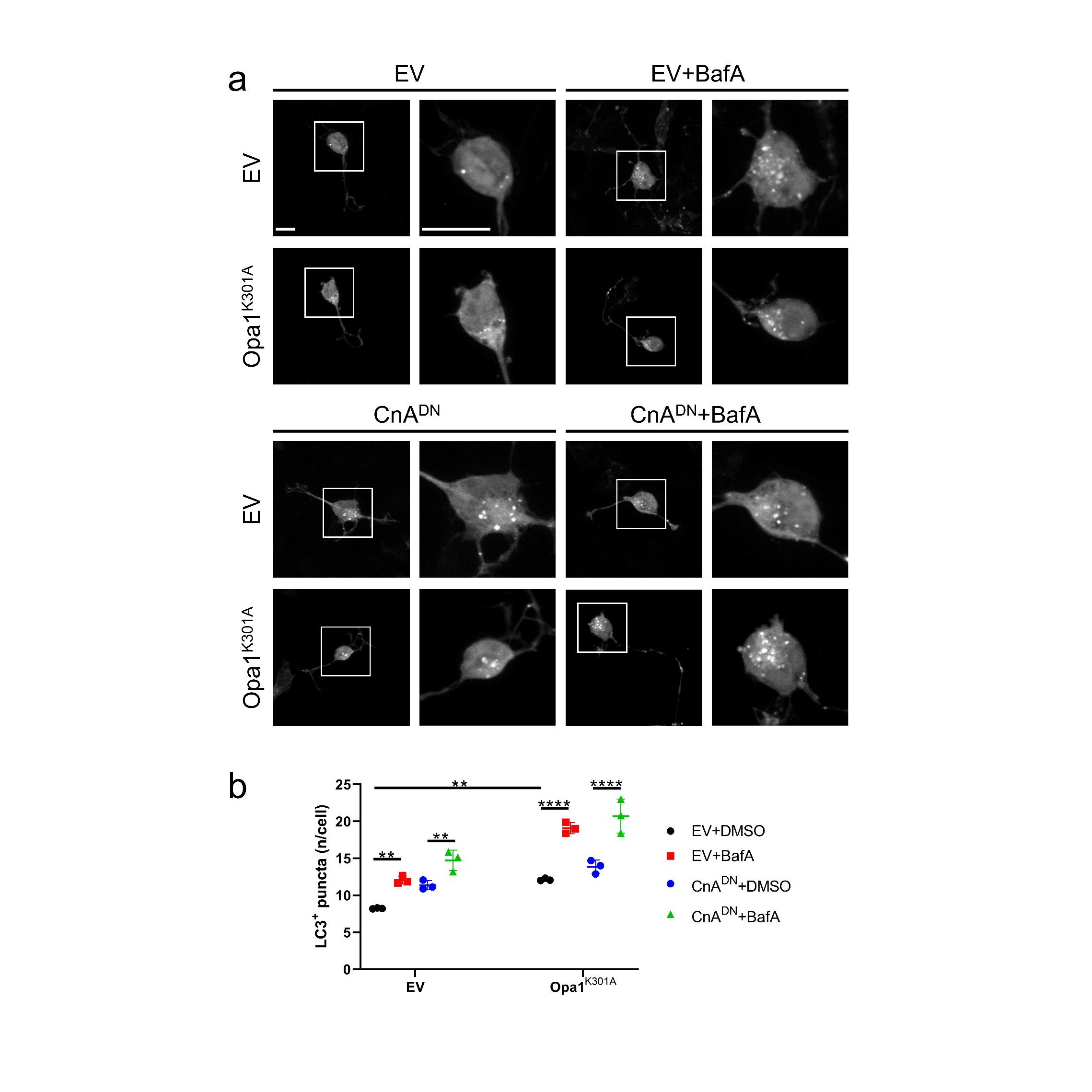
**

**Supplementary Figure 3. Cn^DN^ does not alter autophagic flux in Opa1^K301A^ expressing RGCs.**

**(a)** Representative z-projects of stacks of confocal images of the fluorescence of YFP-LC3 (grey) of primary RGCs co-transfected with the indicated plasmids and treated with Bafilomycin A (BafA). Soma is enlarged in the right panel. Bar, 20 μm.

**(b)** Autophagic flux in experiments as in (a). Data represent average±SD of 3 independent experiments (n=8-19 cells/experiment); ***P*<0.002, *****P*<0.0001; two-way ANOVA with Sidak’s multiple comparison test.

**
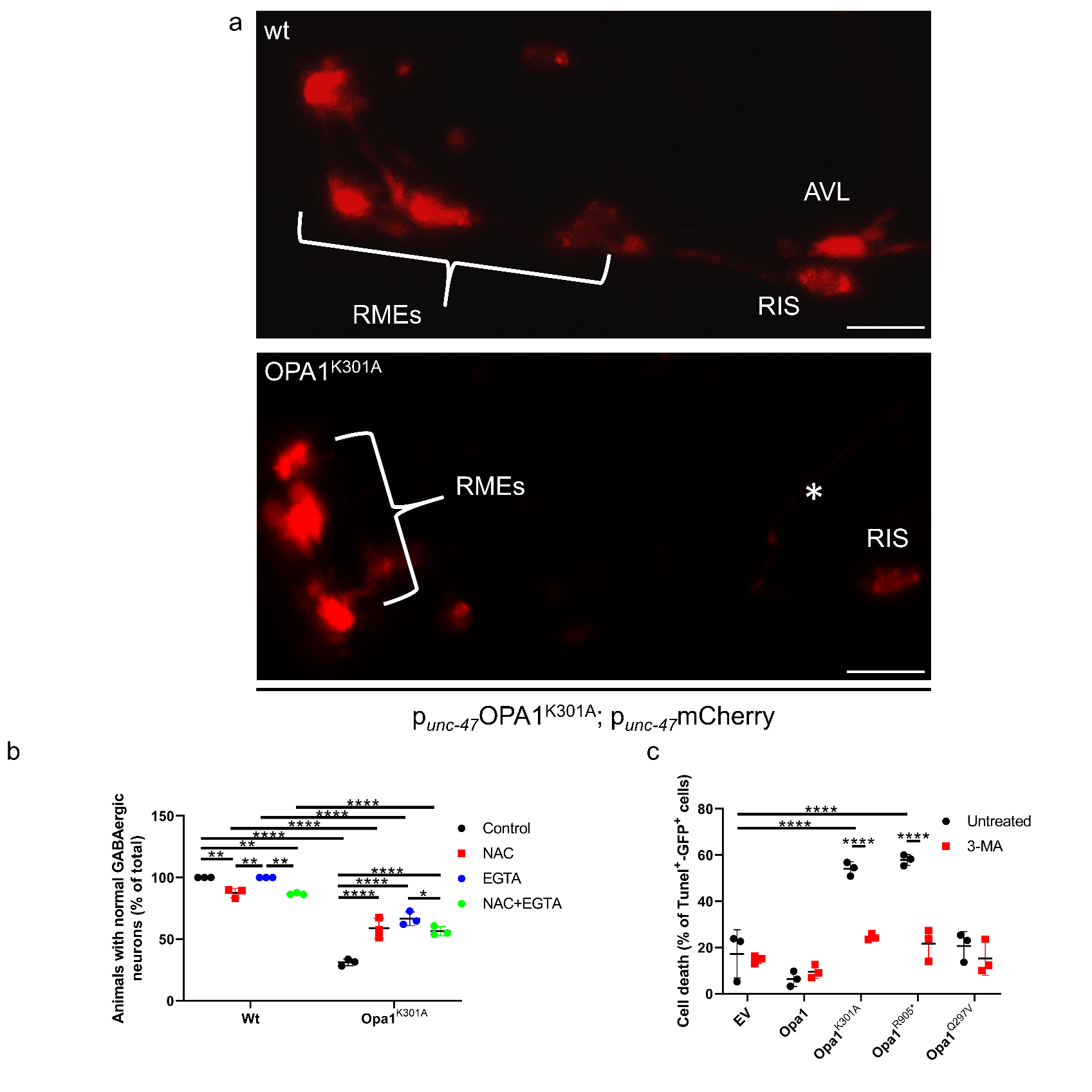
**

**Supplementary Figure 4. Ca^2+^ chelation and autophagy inhibitors blunt neuronal cell death caused by OPA1 pathogenic mutants in nematodes and RGCs.**

**(a)** Representative images of the head region of Wt nematodes and transgenic animals co-expressing OPA1^K301A^ with cytosolic mCherry. The asterisk indicates the loss of GABAergic motor AVL neuron.

**(b)** Experiments were as in **a,** except that where indicated animals were treated with EGTA, NAC or a combination of the two. Data represent average±SD of 3 independent experiments (n=30 animals/experiment); **P*<0.03; ***P*<0.002; *****P*<0.0001; two-way ANOVA with Sidak’s (between genotypes) and Tukey’s (between treatments) multiple comparison tests).

**(c)** RGCs were co-transfected with GFP and the indicated plasmids and treated where indicated with 3-Methyladenine (3-MA). After 72h, apoptosis was measured. Data represent average±SD of 3 independent experiments (n=40 cells/experiment). *****P*<0.0001; two-way ANOVA with Tukey’s (between genotypes) and Sidak’s (between treatments) multiple comparison tests).
